# Fetuin-A Can Assess the Severity of Alcohol-Related Liver Disease

**DOI:** 10.3390/medicina61071147

**Published:** 2025-06-25

**Authors:** Musa Salmanoğlu, İrfan Küçük, Süleyman Baş

**Affiliations:** 1 Department of Internal Medicine, Sultan 2. Abdulhamid Han Training and Research Hospital, University of Health Sciences, Istanbul 34668, Turkey; 2 Department of Gastroenterology, Kartal Dr. Lütfi Kırdar City Hospital, University of Health Sciences, İstanbul 34865, Turkey; drirfn@gmail.com; 3 Department of Internal Medicine, Sancaktepe Sehit Prof. Dr. Ilhan Varank Training and Research Hospital, University of Health Sciences, Istanbul 34785, Turkey; suleymanbas.2012@gmail.com

**Keywords:** alcoholic liver, alcoholic hepatitis, alcoholic cirrhosis, fetuin-A

## Abstract

*Background and Objectives*: Fetuin-A is mostly synthesized in the liver. It is a hepatokine, which is an extracellular inhibitor of growth factors. There is a scarcity of data on the clinical utility of serum fetuin-A (SFA) in alcohol-associated cirrhosis (AC). We first investigated the association between SFA levels and disease phenotypes in alcoholic liver disease (ALD) patients, including alcohol-associated steatotic liver (ASL) and alcohol-associated hepatitis (AH), along with AC patients. *Materials and Methods*: There were 26 healthy controls and 64 ALD patients in this case–control study. The severity of the disease in the AC patients was evaluated using the Child–Pugh classification (CPC-A, -B, and -C), and the FH and AC patients’ Maddrey’s differential function scores and the Model of End-Stage Liver Disease Sodium (MELD-Na) scores were computed. We measured SFA levels using a human fetuin-A enzyme-linked immunosorbent assay (ELISA) kit. *Results*: The SFA concentrations were lower in the AC group and higher in the ASL group [670.72 (412.36) mg/L vs. 1484.61 (858.16) mg/L, respectively; *p* < 0.001]. When compared to patients with ASL, the SFA levels in AC patients were noticeably lower. However, similar SFA levels were observed for the AH group and the healthy controls, as well as for the ASL group and the healthy controls. Within the AC group, the CPC-A subgroup had the highest median SFA values, while the CPC-C subgroup had the lowest median SFA value. Furthermore, the median SFA levels demonstrated significant and inverse correlations with the CPC scores and the MELD-Na scores (rho = −0.671, *p <* 0.001; rho = −0.742, *p <* 0.001, respectively). A negative correlation was observed between the SFA levels and the MELD-Na scores in the AH group (ρ = −0.621, *p* = 0.013). *Conclusions*: In ALD patients, decreased SFA levels, which exhibit disease severity, might be an auxiliary biomarker for the follow-up of AC patients.

## 1. Introduction

Alcohol abuse is a public health care problem that involves psychosocial issues. Alcohol-related liver disease (ALD) has an unclear pathophysiology, and it is a common cause of end-stage liver disease, accounting for approximately 50% of cirrhosis in developed countries [1,2,3]. Alcohol-associated steatotic liver (ASL), steatohepatitis, and alcohol-associated cirrhosis (AC) are consecutive disorders, and 8–20% of patients with steatohepatitis progress to cirrhosis, while AC progresses to hepatocellular carcinoma (HCC) in 3–10% of cases [2]. The determination of the disease severity is mandatory in the follow-up sessions of patients with ALD.

Fetuin-A (alpha 2-heremans-schmid glycoprotein) is a serum glycoprotein that weighs approximately 60 kDa and is mostly synthesized in the liver [4]. It has divergent properties in physiological and pathological conditions, including calcification in bone mineral metabolism [4,5]. In the liver and muscle cells, fetuin-A inhibits insulin receptor tyrosine kinase activity and causes insulin resistance by inhibiting insulin activity [4,5]. It is a negative acute phase reactant and prevents extraosseal calcification, which was exhibited in fetuin-A knockout mice [6]. Fetuin-A is known as a hepatokine, which is an extracellular inhibitor of growth factors, has a profibrogenic stimulus in the liver, and exhibits clinical significance in various liver diseases [7,8,9,10,11,12,13,14].

In the current literature, limited data exist about the diagnostic and prognostic significances of serum fetuin-A (SFA) values in alcohol-related cirrhosis, but there is no information for the other forms of ALD [14,15,16]. Therefore, in this study, we sought to evaluate whether serum fetuin-A levels in ALD patients could serve as a diagnostic and prognostic marker that is associated with the clinical and laboratory phenotypes of the disease.

## 2. Materials and Methods

### 2.1. Study Population

This study was designed as a cross-sectional, single-center investigation. A prospective evaluation was conducted on 64 ALD patients and 26 healthy controls (HCs) who were admitted to our department’s inpatient and outpatient services between July 2024 and January 2025. This study was carried out in accordance with the ethical guidelines specified in the Declaration of Helsinki and was approved by the Ethics Committee of Sancaktepe Sehit Prof. Dr. Ilhan Varank Training and Research Hospital (Approval Code: 2024/175; Approval Date: 28 June 2024). All subjects gave their written informed consent before being included in this study.

Participants were excluded from this study if they had any clinical conditions that could affect their fetuin-A concentrations, including any type of bone disease; disorders of calcium metabolism; severe organ failure, diabetes mellitus; impaired fasting glucose levels, acute or chronic infections; any cancer, including HCC; and seropositive tests for hepatitis B and C viruses. This study excluded volunteers having acute or chronic liver disease of any etiology, including drug-induced liver injury. ALD patients who presented with acute liver failure or hepatic encephalopathy were also excluded from this study.

Male patients consumed excessive alcohol of >60 g per day, and female patients consumed > 40 g per day for at least five years [17]. Some of the patients were referred by the Institute of Alcohol and Substance Use Disorders Research and Treatment Center of the province for a gastroenterology consultation. The *Diagnostic and Statistical Manual of Mental Disorders* was used to identify alcohol use disorder (DSM-5) [18]. The HCs did not use alcohol, and their ultrasonography (USG) reports were normal, with no hepatic steatosis or hepatomegaly. The biochemistry values of the HCs were also in the normal ranges.

### 2.2. Data Collection

For the ALD patients, the dose and duration of alcohol intake were noted. All the participants underwent a USG for the radiologic evaluation, and all the participants needed biochemical tests, including C-reactive protein, before the USG. Based on the disease history, physical findings, laboratory results, and USG results, the ALD patients were classified into three groups, namely alcohol-associated steatotic liver (ASL), alcoholic-associated hepatitis (AH), and alcohol-associated cirrhosis (AC). In the AH patients, the participants’ alcohol intake was continuous for six months or more with <60 days of abstinence before the onset of jaundice, serum aspartate transaminase (AST) values were higher than alanine aminotransferase (ALT) values (AST/ALT ratio > 1.5), serum total bilirubin > 3.0 mg/dL, and without any relevant cause [17].

The AH group was not cirrhotic. AC was diagnosed according to the stigmata of cirrhosis in the participants’ physical examinations and from the results of the biochemical tests and the USG reports. The patients with ASL were not diagnosed with AH or AC. Biochemical tests were obtained prior to USG. In the USG scan, all participants were examined for hepatic steatosis and ascites. The patients with AH and AC underwent an upper gastrointestinal endoscopy, and they were examined to identify any esophageal and/or gastric varices.

### 2.3. Assessment of Disease Severity in Patients with ALD

The Fibrosis-4 (FIB-4) index was used for all participants to predict liver fibrosis [19]. Body mass index (BMI), comorbidities, and medications were recorded for all participants. The scores for Maddrey’s discriminant function (MDF) test and the model for end-stage liver disease sodium (MELD-Na) were calculated in the AH patients [20,21]. For the patients with AC, their Child–Pugh scores (CPS) were calculated, and they were grouped according to their Child–Pugh classification (CPC-A, -B, and -C) [22]. MELD-Na scores were also obtained for the AC patients.

### 2.4. Serum Fetuin-A Measurement

The participants’ venous blood samples were used to obtain the fetuin-A serum. Following a 10 min centrifugation at 30 °C and 5000× *g*, the serum samples in the supernatant were kept in Eppendorf tubes at (−)80 °C until analysis was completed. The participants’ fetuin-A levels were measured using a Human Fetuin-A Enzyme-Linked Immunosorbent Assay (ELISA) kit (Bioassay Technology Laboratory, Cat. No. E1386 Hu, Lot:202309023) (Intra-Assay: CV < 8%, Inter-Assay: CV < 10%) and a microplate reader (Biotech Epoch 2 Microplate ELISA Reader, USA) in accordance with the manufacturer’s instructions

### 2.5. Statistical Analysis

We performed statistical analyses using the Statistical Sciences Package (IBM SPSS, version 27.0) and rstudio (ver. 2024.09.0; pROC package) statistical software. The analysis results were provided as mean ± standard deviation, median, interquartile range (IQR), frequency, and percentage. For the normality of continuous data, the Shapiro–Wilk test was used. To compare the nonparametric data between groups, the Mann–Whitney U test and the Kruskal–Wallis test were used, and to compare parametric data, an independent T-test and ANOVA (post hoc: Bonferroni) were used. To compare the categorical data between groups, a chi-square test was used.

To evaluate the diagnostic value, a receiver operating characteristic (ROC) curve was used, and the accuracy of the fetuin-A molecule between the patient groups. The Youden index was used to determine the cut-off point (J value). Following the determination of the optimal cut-off point, the corresponding diagnostic parameters, namely sensitivity and specificity, were reported. Spearman’s rho correlation analysis was used for the correlation analysis of the parameters that did not follow a normal distribution. A *p*-value < 0.05 was accepted as statistically significant.

## 3. Results

A total of 64 ALD patients (61 males, 3 females) and 26 HCs (21 males, 5 females) participated in this study. The patients’ clinical, laboratory, and demographic details are shown in Table 1. The study population was predominantly male, with statistically significant differences in gender distribution among the groups. Among the ALD patients, those with AC were the oldest (*p* < 0.001). The duration of alcohol consumption was longer in the AC group compared to the AH group. However, there was no significant difference between the ASL and AC groups (Table 1). The HC groups had the lowest mean BMI value, and there was a statistically significant difference in BMI between all the groups. (*p* < 0.001).

The FIB-4 index was similar between the ASL group and the HC group, and it was the highest in the AH group (*p* < 0.001). The median MELD-Na scores were similar between the AC and AH groups. Bilirubin levels were the highest in patients with AH compared to those with ASL and AC, and they were similar between the ASL and HC groups (Table 1). The groups showed a substantial difference in albumin levels, with the HC group having the highest levels and the AC group having the lowest. (*p* < 0.001).

The AC group had the lowest SFA levels, whereas the ASL group had the highest [670.72 (412.36) mg/L vs. 1484.61 (858.16) mg/L, respectively; *p* < 0.001]. Regarding SFA levels, statistically significant differences were found between the AC group and the HC group, between the AC and AH groups, between the AC and ASL patients, and between the AH and ALS. There was no statistically significant difference between the AH group and the HC group, nor between the ASL group and the HC group (Table 1).

In both the AC and AH groups, there was no statistically significant difference in SFA levels between the patients with or without steatosis nor between those with or without ascites (Table 2). Within the AC group, patients without upper gastrointestinal varices exhibited higher median SFA concentrations compared to those with varices [812.95 (536.33) mg/L vs. 603.35 (393.2) mg/L, respectively; *p* = 0.026]. In the AH group, the difference in the median SFA values between the patients with and without upper gastrointestinal varices was not statistically significant.

According to the CPC, there was a statistically significant difference among the classes in the AC group (*p* = 0.002). The patients in the CPC-A subgroup had the highest median SFA values, whereas those in the CPC-C subgroup had the lowest median SFA values (Table 2). There was no significant difference between the SFA levels of the CPC-A and CPC-B subgroups, but the SFA levels were lower in the CPC-C subgroup than in the CPC-A and CPC-B subgroups (*p* = 0.091; *p* = 0.003, *p* = 0.012, respectively) (Table 2).

In the patients with ASL, no statistically significant correlations were observed between the SFA concentrations with C-reactive protein (CRP) levels and the FIB-4 index. Similarly, in the AH group, no significant correlations were found between the SFA levels with CRP and the MDF values. However, there was a negative correlation between the SFA levels and the MELD-Na scores in the AH group (ρ = −0.621, *p* = 0.013) (Table 3). In the patients with AC, significant and inverse correlations were observed between the median SFA levels and the CRP concentrations, CPC scores, and MELD-Na scores (ρ = −0.569, *p* = 0.002; ρ = −0.671, *p* < 0.001; ρ = −0.742, *p* < 0.001, respectively).

**Figure 1:**
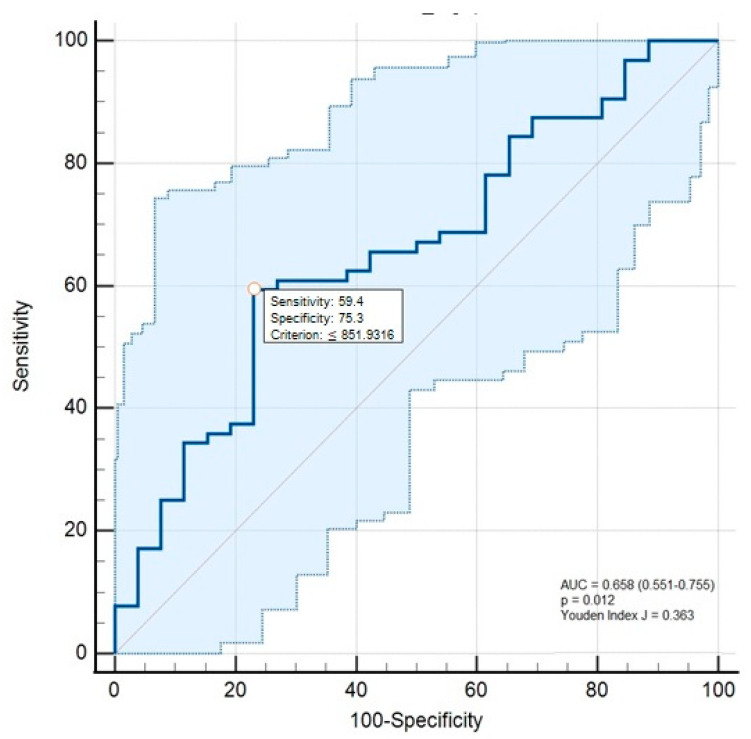
Receiver operating characteristics curve analyses of the serum fetuin-A concentrations for the differentiation of alcohol-associated cirrhosis from healthy controls.

The ROC curve analyses revealed that the optimal cut-off point for SFA concentrations was 851.93 mg/L, with a sensitivity of 59.4% (95% CI: 46.4–71.5) and a specificity of 75.3% (95% CI: 56.3–91.0). These results suggest the potential diagnostic performance and the utility of SFA in prognostic applications (Figure 1).

## 4. Discussion

High SFA levels have been declared to have diagnostic and prognostic values for metabolic dysfunction-associated steatotic liver (MASLD), type 2 diabetes, and atherosclerosis [4,5,12].

Low SFA levels were measured in autoimmune hepatitis, primary biliary cholangitis, drug-induced hepatitis, and hepatocellular carcinoma [8,9,10]. However, the SFA values did not change in the follow-up acute A, B, or Epstein–Barr virus hepatitis, and a paradoxical increase was noted in chronic C virus hepatitis [11,15]. Despite the diabetogenic effect of fetuin-A in MASLD, moderate alcohol intake is known to be associated with lower circulating fetuin-A concentrations, which act as an insulin sensitizer [12,14,23]. In the cohort of diabetes-free women, moderate alcohol consumption was declared to be associated with lower plasma fetuin-A levels, and participants using higher amounts of daily alcohol had lower circulating fetuin-A values than those using lower amounts of daily alcohol [14].

In our results, the BMI of the AH patients was the highest, whereas it was the lowest in the control group. In the AH group, excessive and continuous alcohol ingestion can lead to obesity, hepatic steatosis, and insulin resistance (IR). In large cohorts, elevated SFA concentrations were declared to have a risk for type-2 diabetes, and a possible mechanism for diabetes was proposed as IR caused by the inhibition of insulin signaling with fetuin-A, and fetuin-A has emerged as a biomarker for diabetes risk [12,14]. In the cohort of diabetes-free women, moderate alcohol consumption was declared to be associated with lower plasma fetuin-A levels, and participants using higher daily alcohol had lower circulating fetuin-A values [14]. Although the higher BMI values were noted in the ALD patients, they had lower SFA concentrations compared to the HCs, and as noted earlier, and in light of current data, this result may be possibly due to the insulin-sensitizing effect of ethanol, but further investigations are needed to confirm [12,14,23]. In the current study, the participants with diabetes mellitus and impaired fasting glucose levels were not enrolled to exclude the alterations of SFA concentrations due to glucose metabolism in the ALD patients. As a limitation, we did not detect insulin values in our cohort, and measurement of the fasting insulin values could reveal the relationship between fasting insulin values and SFA concentrations in the ALD patients.

In our cohort, the duration of alcohol ingestion was higher in the AC patients than in the patients with AH, and it was similar in the ALS and AC patients. A long duration of ethanol use can exert severe liver injury and fibrogenic effects seen in AC patients, as expected [2]. One-fifth of alcohol abusers can progress to end-stage liver disease, and it is an enigma to predict which patients may develop severe disease due to unresolved pathogenic mechanisms in ALD [1,2,3]. This may partly explain the similar duration of ethanol ingestion in the ASL and AC patients. According to the Fib-4 index formula, the highest median Fib-4 index in the AH group can be ascribed to elevated AST values in AH [17,19]. On the other hand, patients with AH usually present with jaundice and elevated bilirubin values due to acute and subacute liver injury, and this may be a possible reason for the elevated bilirubin values in the AH patients compared to other study groups [17]. With respect to SFA concentrations, no statistically significant differences were found between the AH group and the HC group nor between the ASL group and the HC group, but larger sampled-sized cohorts are needed for further evaluation. We reported that SFA concentrations were the lowest in the AC patients compared to ASL, AH patients, and HCs and that they were similar between the AH group and the HC group and between the ALS and HCs. Prystupa et al. also found lower concentrations of circulating fetuin-A in AC patients compared to the control group [16]. Additionally, Kalabay et al. noted that baseline lower SFA levels in AC patients were associated with higher mortality rates than those with higher fetuin-A values at one-year follow-up sessions [15].

For the AC group, our results highlight the diagnostic utility of SFA, as was declared in previous reports, and that low SFA values can have diagnostic utility in AC patients [15,16]. However, we did not include cirrhotic patients in the AH group, which might be a contributing factor for the similar serum fetuin-A values between the AH patients and HCs. In the study of Kalabay et al., baseline mean serum fetuin-A levels of AC patients with concomitant AH, a small percentage of the cohort, were similar to those without AH [15]. The researchers detected increased fetuin-A concentrations in the AC patients with AH during the follow-up period [15]. The diagnostic significance of SFA in ALS and AH patients may be a subject of future research.

Fetuin-A is a negative acute phase reactant [13]. Previously, it was stated that low SFA concentrations could be the result of reduced fetuin-A synthesis due to hepatocyte dysfunction rather than acute phase reaction in AC patients [15,24]. According to our results, the median CRP concentration was the lowest in the HC group. CRP levels were found to be higher in the AH group compared to the ASL patients but were similar between the AH and AC groups. In the AC patients, an inverse and statistically significant correlation was observed between CRP and fetuin-A. However, no such correlation was detected in the ASL and AH groups.

To exclude the alterations due to inflammation, participants with over-acute/chronic infections were not enrolled in our cohort. Ethanol disrupts the intestinal barrier, and gut-derived endotoxins, alcohol metabolites, and activation of innate immunity may lead to alcohol-associated chronic inflammation in AC [1,3]. Higher CRP values in the AC group compared to the HC group may be a representative of this situation. Although there is limited data evaluating the significance of circulating fetuin-A values in ALD patients, in addition to the hepatocyte dysfunction, it is possible that fetuin-A acts like a negative acute phase reactant in AC according to our results, but it needs to be further elucidated.

Although CPC, MELD-Na, and MDF are the quantitative scores that are widely used in practice for the assessment of disease severity and especially for therapeutic options, they have some drawbacks [20,21,22,25]. CPC includes subjective variables such as ascites and encephalopathy, and extrahepatic disorders can alter the variables used in the MELD-Na calculation, and the results of these formulations may not always be parallel to each other [22]. In the study of Kalabay et al., SFA levels, Child–Pugh, and MELD-Na scores were calculated at the time of enrollment and at the 1st, 3rd, 6th, and 12th months thereafter in AC patients [15]. In that study, SFA levels were declared to be superior to CPC and MELD-Na scores for predicting mortality at the follow-up interval, and the authors proposed the serum fetuin-A levels as an independent predictor of long-term mortality [15]. Regarding our results, inverse and significant correlations between the CPC and MELD-Na scores and SFA levels can support the idea that SFA values may serve as objective auxiliary biomarkers for the evaluation of disease severity in the follow-up of AC patients.

The AC patients with ascites had lower SFA levels, but it did not show statistical significance in our results. On the other hand, the cirrhotic patients with upper gastrointestinal varices had significantly lower SFA values. It is noteworthy that the AC patients in the CPC-C subgroup had lower SFA levels than those in the CPC-A and CPC-B subgroups. Although CPC-B patients might have a worse prognosis than those in the CPC-A subgroup, in both subgroups, AC patients can present with compensated disease. Therefore, this result might explain the similar SFA levels in the CPC-A and CPC-B subgroups. However, as a limitation, the number of participants was low in our study. We detected inverse correlations between the SFA concentrations and the CPC and the MELD-Na scores in the AC patients. Within the AC group, patients without upper gastrointestinal varices exhibited higher median SFA concentrations compared to those with varices [812.95 (536.33) mg/L vs. 603.35 (393.2) mg/L, respectively; *p* = 0.026]. In the AH group, the difference in median SFA values between patients with and without upper gastrointestinal varices was not statistically significant. On the other hand, lower SFA concentrations with upper gastrointestinal varices can point out the increased portal pressure that is associated with worse clinical outcomes in AC patients. All these results highlight the prognostic significance of SFA in AC. In the AH group, there was also an inverse correlation between the SFA values and MELD-Na scores, and large sample-sized studies may delineate the diagnostic performance of SFA in AC.

Prystupa et al. also declared that the AC patients in the CPC-C group had the lowest circulating fetuin-A levels [16]. However, in that study, correlations between the MELD-Na and fetuin-A were not evaluated. Although the Fib-4 index was reported to have a predictive value for hepatocellular carcinoma in ALD patients, we did not find any correlation between the fetuin-A values and the Fib-4 index. This may be partly due to the fact that we did not include any participants with malignancy, including HCC [26].

This study’s main limitation is the comparatively small size of the population, as this study is a single-center study. Although the female gender is more prone to alcohol-associated liver injury compared to the male gender, ALD is more prevalent in males [2]. Thus, we could not include more female patients in the patient groups. This is also another limitation of our study. With respect to gender, a comparison of the SFA levels of the male and female patients could reveal valuable information. Performing a liver biopsy could be more valuable for the diagnosis of ALD, and the detection of tissue expression of fetuin-A could also be more precise, but a biopsy was not performed because of its invasive nature and ethical considerations.

## 5. Conclusions

In patients with AC, serum fetuin-A concentrations may hold diagnostic and prognostic significance, serving as an auxiliary biomarker during follow-up sessions. To assess the diagnostic accuracy of fetuin-A, further studies with larger cohorts are required, as well as their relationship with the clinical and laboratory characteristics of ALD.

## Figures and Tables

**Table 1:** Clinical, demographic, and laboratory characteristics and serum fetuin-A values in patients with alcoholic liver diseases and healthy controls.

	Alcohol-Associated Steatotic Liver Group (*n* = 22)	Alcohol-Associated Hepatitis Group (*n* = 15)	Alcohol-Associated Cirrhosis Group (*n* = 27)	Healthy Control Group (*n* = 26)	*p*
Male, *n* (%)	22 (100) ^c^	14 (93.33)	25 (92.59)	21 (77.78) ^c^	<0.001 ^3,^*
Age (years), mean ± SD	48.41 ± 9.54 ^e^	47.07 ± 8.84 ^d^	60.3 ± 7.51 ^d,e^	55.26 ± 11.5	<0.001 ^1,^*
Duration of alcohol intake (years), median (IQR)	25.5 ± 12.18	24.13 ± 10.2 ^d^	36.59 ± 12.79 ^d^	-	<0.001 ^2,^*
BMI (kg/m^2^), mean ± SD	28.48 ± 2.58 ^e^	30.8 ± 4.07 ^b,d^	28.9 ± 3.73 ^d,e^	26.01 ± 3.72 ^b^	<0.001 ^1,^*
Presence of hepatic steatosis, *n* (%)	22 (100) ^e^	14 (93.3) ^d^	4 (14.8) ^d,e^	-	<0.001 ^3,^*
Presence of ascites, *n* (%)	-	6 (40) ^d^	12 (44.44) ^d,e^	-	<0.001 ^3,^*
Presence of esophageal and/or gastric varices, *n* (%)	0 (0) ^e,f^	3 (20) ^d,f^	16 (59.3) ^d,e^		<0.001 ^3,^*
Fibrosis-4 index, median (IQR)	1.23 (0.89) ^e,f^	6.46 (5.06) ^b,d,f^	5.38 (3.56) ^a,d,e^	1.13 (0.46) ^a,b^	<0.001 ^2,^*
MELD-Na score, median (IQR)	-	16.2 ± 6.56	14.71 ± 7.42	-	0.401 ^2^
MDF, median (IQR)	-	14.90 (17.2)	-	-	-
Child–Pugh class, *n* (%) A B C	-	-	10 (37.04) 8 (29.63) 9 (33.33)	-	N/A
Child–Pugh score, median (IQR)	-	-	7.96 (5)	-	N/A
Bilirubin (mg/dl), median (IQR)	0.67 (0.44) ^e,f^	4.49 (2.11) ^b,d,f^	2.36 (2.1) ^a,d,e^	0.73 (0.61) ^a,b^	<0.001 ^2,^*
Albumin (mg/dl), median (IQR)	4.30 (0.65) ^c,f^	3.85 (0.90) ^b,d,f^	3.16 (1.80) ^a,d^	4.62 (0.65) ^a,b,c^	<0.001 ^2,^*
CRP (mg/L), median (IQR)	4.95 (10.36) ^c,f^	13.27 (24.32) ^b,f^	7.08 (18.62) ^a^	1.38 (2.64) ^a,b,c^	<0.001 ^2,^*
Serum fetuin-A (mg/L), median(IQR)	1484.61 (858.16) ^e,f^	906.75 (383.95) ^d,f^	670.72 (412.36) ^a,d,e^	1553.95 (692.8) ^a^	<0.001 ^2,^*

Abbreviations: IQR: interquartile range; SD: standard deviation; BMI: body mass index; MELD-Na: model for end-stage liver disease sodium; MDF: Maddrey’s discriminant function; CRP: C-reactive protein. * Statistically, the significance level was lower than 0.05. According to the pairwise comparison, it was different from healthy controls vs. alcohol-associated cirrhosis ^a^, healthy controls vs. alcohol-associated hepatitis ^b^, healthy controls vs. alcohol-associated steatotic liver ^c^, alcohol-associated cirrhosis vs. alcohol-associated hepatitis ^d^, alcohol-associated cirrhosis vs. alcohol-associated steatotic liver ^e^, alcohol-associated hepatitis vs. alcohol-associated steatotic liver ^f^. ^1^ ANOVA test (post hoc: Bonferroni). ^2^ Kruskal–Wallis test (post hoc: Mann–Whitney U Test). ^3^ Chi-Square test.

**Table 2:** Serum fetuin-A concentrations and clinical and laboratory variables in patients with alcoholic hepatitis and cirrhosis.

				Serum Fetuin-A (mg/L)	
			*n*	Median (IQR)	*p*
Alcohol-associated hepatitis (*n* = 15)	Hepatic steatosis	Absent	1	637 (N/A)	N/A
		Present	14	826.58 (365.97)	
	Ascites	Absent	9	822.06 (379.36)	0.724 ^1^
		Present	6	824.5 (384.21)	
	Esophagus and/or gastric varices	Absent	12	885.48 (393.26)	0.386 ^1^
		Present	3	700.41 (N/A)	
Alcohol-associated cirrhosis (*n* = 27)	Hepatic steatosis	Absent	23	698.11 (383.50)	0.733 ^1^
		Present	4	767.29 (669.50)	
	Ascites	Absent	15	700.71 (349.1)	0.143 ^1^
		Present	12	498.25 (510.24)	
	Esophagus and/or gastric varices	Absent	11	812.95 (536.33)	0.026 ^1^
		Present	16	603.35 (393.2)	
	Child–Pugh Classification	A ^a^	10	844.71 (412.31)	0.002 ^2,^*
		B ^b^	8	740.52 (293.8)	
		C ^a,b^	9	356.82 (325.21)	

* Statistically, the significance level was lower than 0.05. According to the pairwise comparison, it was different from Child–Pugh Classification A vs. Child–Pugh Classification A ^a^, and Child–Pugh Classification B vs. Child–Pugh Classification C ^b^ (pa = 0.003; pb = 0.012). ^1^: Mann–Whitney U Test, ^2^: Kruskal–Wallis test (post hoc: Mann–Whitney U test).

**Table 3:** Correlations between the serum fetuin-A values and the laboratory and clinical variables of the patients with alcoholic liver diseases.

		Serum Fetuin-A				
		CRP	Child–Pugh Score	MELD-Na	MDF	Fibrosis-4 Score
Alcohol-associated steatotic liver (*n* = 22)	rho	−0.121	-	-	-	0.193
	*p*	0.590	-	-	-	0.389
Alcohol-associated hepatitis (*n* = 15)	rho	0.021	-	−0.621 *	−0.399	0.042
	*p*	0.940	-	0.013 *	0.141	0.881
Alcohol-associated cirrhosis (*n* = 27)	rho	−0.569 *	−0.671 *	−0.742 *	-	−0.330
	*p*	0.002 *	<0.001 *	<0.001 *	-	0.092

Abbreviations: MELD-Na: model for end-stage liver disease sodium; MDF: Maddrey’s discriminant function. * Correlation is significant at the 0.05 level.

## Data Availability

The dataset is available upon request from the authors.
